# Retrospective Analysis of Bone Metabolism in Patients on Waiting List for Simultaneous Pancreas-Kidney Transplantation

**DOI:** 10.1155/2019/5143021

**Published:** 2019-05-14

**Authors:** Simona Kratochvílová, Jana Brunová, Petr Wohl, Věra Lánská, František Saudek

**Affiliations:** Diabetes Center, Institute for Clinical and Experimental Medicine, Prague 140 21, Czech Republic

## Abstract

Posttransplant osteoporosis, which evolves from preexisting bone pathologies, represents a serious complication with deteriorating consequences. The aim of our study was to evaluate epidemiological data on bone mineral density (BMD) in subjects with type 1 diabetes (T1DM) in advanced stages of diabetic nephropathy indicated for simultaneous pancreas-kidney transplantation (SPK). We retrospectively compiled biochemical and densitometrical data from 177 patients with T1DM at CKD (chronic kidney disease) stages G4-G5 (115 men, 62 women, median age 40 yr, diabetes duration 23 yr) enrolled on waiting list for SPK for the first time between the years 2011 and 2016. Median *Z*-scores were as follows: lumbar spine (LS): -0.8 [interquartile range -1.75 to 0.1]; total hip (TH): -1.2 [-1.75 to -0.6]; femoral neck (FN): -1.2 [-1.9 to -0.7]; and distal radius (DR): -0.8 [-1.4 to -0.1]. We noted a gender difference in LS, with worse results for men (-1.1 vs. -0.3) even after adjusting for BMI (body mass index) and glomerular filtration (*p* < 0.001). Osteoporotic and osteopenic ranges (based on *T*-scores) for all major sites were 27.7% and 56.5%, respectively, with similar results across both genders. Women had a significantly higher proportion of normal BMD in LS than men (67.7 vs. 49.4%, *p* < 0.05). Patients with T1DM at CKD stages G4-G5 exhibited serious BMD impairment despite their young age. Men surprisingly displayed lower *Z*-scores and higher percentages of pathological BMD values in LS than women did. The introduction of adequate preventive measures during the advanced stages of diabetic nephropathy to prevent bone loss is recommended.

## 1. Introduction

Type 1 diabetes is an autoimmune disease with lifelong insulin dependence, and despite the fact that great progress has been made in implementing modern ways of treatment (insulin analogues, continuous subcutaneous insulin infusions, and continuous glucose monitoring systems) into the everyday glucose management, patients still face the risk of glucose excursions with consequent development of late complications. All tissues and organs may be targeted by diabetes, and besides the major well-known microvascular (retinopathy, nephropathy, and neuropathy) and macrovascular (atherosclerosis) complications, type 1 diabetes mellitus is associated with low bone mineral density (BMD) and increased risk of fracture [1]. Its negative impact on bones is mediated through the deficient anabolic effect of insulin, the toxic effect of hyperglycaemia, the accumulation of advanced glycation end products in the bone matrix, oxidative stress, inflammation, microangiopathy, and excessive urinary calcium excretion [1–3]. Furthermore, diabetic nephropathy affects approximately one-third of subjects with diabetes and can eventually progress to end-stage renal disease (ESRD) requiring renal replacement therapy. According to a recent analysis, 30-year cumulative incidence of ESRD among patients with type 1 diabetes ranged from 3.3 to 7.8% [4]. The gradual deterioration of renal function is accompanied by bone impairment due to hypocalcaemia, vitamin D deficiency, and secondary hyperparathyroidism. In cases where unprevented chronic kidney disease-associated metabolic bone disease (CKD-MBD) is established, subjects with preceding diabetic osteopathy are at extreme risk of developing profound bone loss.

Tight glycemic control is the best prevention of late complications as demonstrated in the DCCT/EDIC trial [5]. Pancreas transplantation is a functional and effective therapy that restores endogenous insulin secretion and enables achieving stable normoglycemia [6]; islet transplantation offers a less invasive alternative [7]. Simultaneous pancreas and kidney transplantation (SPK) is an established treatment option for subjects with type 1 diabetes and renal failure [8]. The major benefits of SPK are improved quality of life and life expectancy [9, 10]. Whereas multiple positive effects such as restored renal function, stabilization, or even improvement of diabetic retinopathy [11–13] or neuropathy [14, 15] and reduction in macrovascular disease [16–21] have been reported after the successful transplantation, ongoing decline in bone mineral density and increased number of fractures is often encountered. According to a recent study, 35.4% of subjects after SPK were categorised as having osteoporosis in the lumbar spine, with 39.6% in the femoral neck [22]. The incidence of fractures is very high following SPK, even in comparison with other solid organ recipients [23]. Etiology of posttransplant osteoporosis is multifactorial comprising the negative impact of immunosuppressive agents, persistent parathyroid disease, and vitamin D deficiency [24], but preexisting bone pathology plays a crucial role in its development [25].

The need for implementing effective preventive measures during the pretransplant period is critical. However, the epidemiological data on bone health specifically for subjects on waiting list for SPK or with type 1 diabetes in advanced stages of diabetic nephropathy are still scarce.

Subjects with type 1 diabetes mellitus form only a relatively minor part of all diabetic patients suffering from renal failure, and in studies published thus far, they tend not to be distinguished from type 2 diabetes patients with different bone impairment pathogenesis [26]. Moreover, up until recently, BMD was not considered predictive of fracture risk in CKD patients. But in light of the most recent KDIGO guideline update in 2017 [27], this assumption is now being challenged. It is now acknowledged that BMD predicts fractures even in CKD.

The aim of our study was to evaluate BMD and bone metabolism in all patients enrolled on waiting list for SPK for the first time in the Czech Republic between the years 2011 and 2016. Our SPK programme is well-established, recently averaging 3.7 pancreas transplantations per million of the population, and was the third most active worldwide in 2017 according to the IRODAT registry [28]. All type 1 diabetes patients suffering from CKD G4-G5 are referred to our center and considered for suitability. Bone metabolism screening (bone densitometry and laboratory parameters) forms an integral part of the pretransplant examination. The timely assessment of risk factors enables preventive strategies to be formulated for patients in the earlier stages of diabetic nephropathy.

## 2. Materials and Methods

Between the years 2011 and 2016, 182 patients with type 1 diabetes were enrolled on waiting list for their first SPK transplantation. Eligibility criteria were as follows: age under 65, CKD G4-G5 (patients on haemodialysis, peritoneal dialysis, or predialysis patients with a glomerular filtration rate under 30 mL/min/1.73 m^2^), absence of cardiac failure, advanced cardiovascular disease, malignity, infection, or any other evident contraindication. The following 6 exceptions applied to primary diagnosis: 4 subjects had maturity onset diabetes of the young (MODY 1 and 3) confirmed by genetic testing, while 2 others were confirmed with acute necrotising pancreatitis after total or subtotal pancreatectomies. CKD of combined etiology was only confirmed in two women with systemic lupus erythematosus and anti-neutrophil cytoplasmic antibody (ANCA) vasculitis. Seven men were placed under pretransplant investigation at stage CKD G3b.

Data were retrospectively retrieved from medical documentation corresponding to the first pretransplant investigations performed between the years 2010 and 2016. We analysed epidemiological characteristics (age, age of diabetes onset, and disease duration), anthropometric parameters (body mass index, BMI), medical history, treatment, biochemical results, and densitometric parameters. Time-relevant densitometry was not available in the case of 5 subjects, which explains why the final analysis totalled 177 patients. The study was approved by the local ethics committee.

### 2.1. Laboratory Measurements

Total serum calcium, phosphate (s-P), creatinine, urea, albumin, and total serum alkaline phosphatase (ALP) were analysed spectrophotometrically using automated analysers, while intact parathyroid hormone (PTH) was measured by electrochemiluminescence immunoassay. Levels of vitamin D (25OHD) and calcitriol (1,25(OH)_2_D_3_) were measured by the radioimmunoassay method (using kits from DIAsource Immunoassays S.A., Louvain-la-Neuve, Belgium, and IDS Immunodiagnostic Systems, Boldon, UK, respectively) and glycosylated haemoglobin (HbA1c) by high-performance liquid chromatography, as calibrated to the IFCC reference procedure [29]. Both IFCC and calculated DCCT values are presented. The estimated glomerular filtration (GF) rate was calculated from the MDRD formula [30]. Total serum calcium was corrected to serum albumin levels, where s − Ca = total serum calcium + 0.02*x* (41.3-albumin).

Vitamin D (25OHD) status was defined according to K/DOQI guidelines [31] (<5 ng/mL severe deficiency, 5-15 ng/mL mild deficiency, and 16-30 ng/mL insufficiency). Our laboratory standard for calcitriol was 19.6-54.3 ng/L. Optimal concentrations of s-Ca, s-P, and PTH for respective CKD stages were classified according to K/DOQI guidelines [31] (s-P CKD G4: 0.87-1.48 mmol/L, CKD G5: 1.11-1.78 mmol/L, s-Ca: normal laboratory range 2.15-2.55 mol/L, PTH CKD G4: 7.7-12.1 pmol/L, and CKD G5: 16.5-33 pmol/L). A complete set of s-Ca, s-P, and PTH blood values was available for 173 patients. Vitamin D concentrations were available for 148 patients and calcitriol concentrations for 147 patients.

### 2.2. Bone Densitometry

All subjects underwent bone densitometry (DXA) of the L1-L4 lumbar spine (LS), total hip (TH), femoral neck (FN), and distal radius (DR). BMD was estimated by dual-energy X-ray absorptiometry on an apparatus (Lunar Prodigy Primo, GE Healthcare Lunar, Madison, WI, USA) used throughout the whole study period. Instrument quality control on the DXA scanner was performed daily using a standard spine phantom. The coefficients of variation for BMD measurements were 1%, both for the spine and the total hip. The values were evaluated using enCORE software, version 13.60.033 (GE Healthcare, Madison, WI, USA) with USA Combined NHANES/Lunar reference population. Results were expressed in absolute values (g/cm^2^) as gender-specific *T*-scores (standard deviation from the mean BMD for a young healthy population) and *Z*-scores (standard deviation from the mean BMD for a population of the same age). We used the World Health Organization criteria [32] to define osteoporosis (*T*-score≤−2.5 SD) and osteopenia (*T*-score<−1 and >−2.5 SD). DR BMD results were missing for 16 subjects.

### 2.3. Statistical Analysis

Values are reported as medians [25^th^ to 75^th^ interquartile ranges] due to the nonnormal distribution of most variables. Categorical variables are presented as the number and percentage of subjects. To compare the study subgroups, the Student *t*-test or Mann-Whitney test was used depending on the distribution. Adjustment for age, BMI, and GF was used where appropriate using the analysis of covariance test. The Friedman test was performed to compare the BMD of particular skeletal sites, while differences in the prevalence of osteoporosis and osteopenia between men and women were tested using the chi-square test. Spearman's correlation coefficient was used to evaluate associations between anthropometric, laboratory, and osteological parameters, with partial correlations used to exclude the effect of confounding variables. Stepwise multiple linear regression analysis (with BMD values as dependent variables) was applied to explain the effects of anthropometric and laboratory parameters on BMD variance. All statistical tests were two-tailed, with *p* ≤ 0.05 considered statistically significant. GraphPad Prism 5 (GraphPad Software, La Jolla, CA, USA) and JMP 11 (SAS Institute Inc., Cary, NC, USA) statistical software were used for data analysis.

## 3. Results

### 3.1. Anthropometric and Laboratory Characteristics

Anthropometric and laboratory characteristics are listed in Table 1. Men dominated in our study (*n* = 115; 65%) and were slightly older than women. Although both gender groups differed with regard to age of diabetes onset, disease duration was the same. Men had higher BMIs, but diabetes control according to HbA1c was similar. More than one-third (*n* = 65; 37%) of patients had previously been on haemodialysis (*n* = 50) or peritoneal dialysis (*n* = 15). The median duration of dialysis treatment in those concerned was 6 [3-14.5] months. In total, women had moderately lower GF rates; however, we list GF values separately for each functional category due to interpretation bias in dialysed subjects. Biochemical parameters were comparable between men and women except for albumin, which was lower in women. Hypercalcaemia was detected in 19 (10.8%) subjects, with only one case of hypocalcaemia. Hyperphosphataemia was found in 73 (41.5%) subjects, with hypophosphataemia observed in 3 (1.7%) subjects. Levels of PTH were of a similar proportion under (*n* = 61; 35%) and above (*n* = 55; 31.6%) the desired limit for the respective renal function. Only 34 (19.7%) subjects had all of their values within the recommended range. The median vitamin D concentration was comparable between men and women. Of the 148 subjects with known concentrations, 0 exhibited severe deficiency, 91 (61.5%) exhibited mild deficiency, and 48 (32.4%) had insufficient levels, while only 9 subjects (6.1%) had normal vitamin D concentrations. Calcitriol concentrations were undetectably low in 27 (18.4%) patients, and of the remaining 120 values, 74 (50.3%) were below the limit and 46 (31.3%) within the normal range.

### 3.2. Densitometric Parameters

BMD values of LS, TH, FN, and DR (expressed as *T*-scores, *Z*-scores, and BMD in g/cm^2^) are listed in Table 2. Median *T*- and *Z*-scores<−1 SD were registered in TH and FN in all subjects and in LS in men. There was a statistically significant difference in *Z*-scores between the examined bone sites (LS, TH, FN, and DR), with TH and FN the most affected areas (*p* < 0.001). In terms of gender comparison, men displayed significantly lower *T*- and *Z*-scores in LS than women, a difference that remained significant even after adjustments for age, BMI, GF, and albumin (*p* < 0.001). BMD absolute values (g/cm^2^) should have been lower in women in all sites, but this applied only to TH and DR.

**Figure 1:**
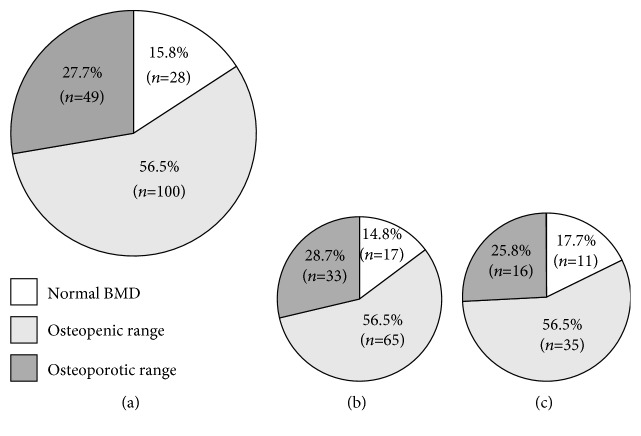
Total prevalence of normal BMD and osteopenic/osteoporotic range in the whole group, in men and in women. (a) The whole group, *n* = 177. (b) Men, *n* = 115. (c) Women, *n* = 62.

**Figure 2:**
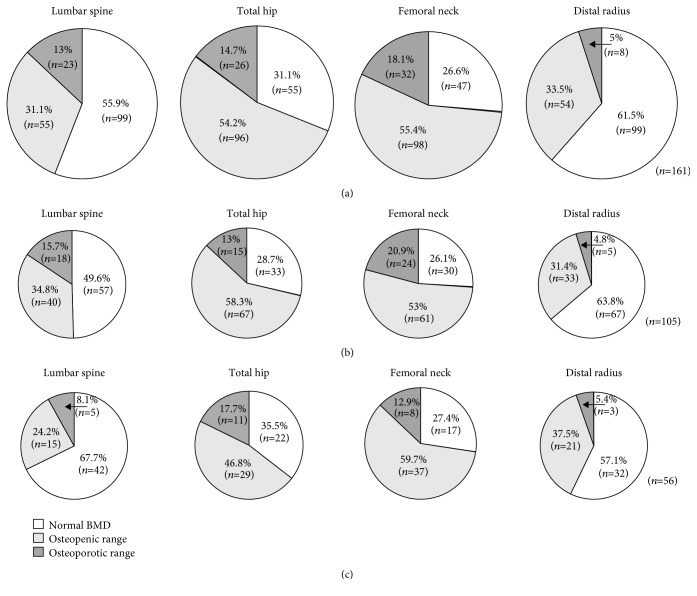
Prevalence of normal BMD and osteopenic/osteoporotic range for all major sites in the whole group, in men and in women. (a) The whole group, *n* = 177. (b) Men, *n* = 115. (c) Women, *n* = 62.

The number and percentage of subjects to meet the osteoporosis criteria in general and particular areas for both men and women are listed in Figures 1 and 2. BMD within the osteoporotic range (in at least one of the major sites) applied to 49 (27.7%) subjects and within the osteopenic range to 100 (56.5%), while only 28 (15.8%) subjects had normal BMD. The proportion was similar between men and women. When comparing individual areas, the prevalence of osteoporosis, osteopenia, and normal BMD differed (*p* < 0.001); the highest occurrence of pathological values was for TH and FN, while DR seemed to be the most spared area. In terms of gender comparison, the prevalence of individual BMD categories in particular bone sites was similar between men and women; only in the LS site did women display a significantly higher proportion of normal BMD than men (42/62 vs. 57/115; 67.7 vs. 49.6%; *p* < 0.05).

**Figure 3:**
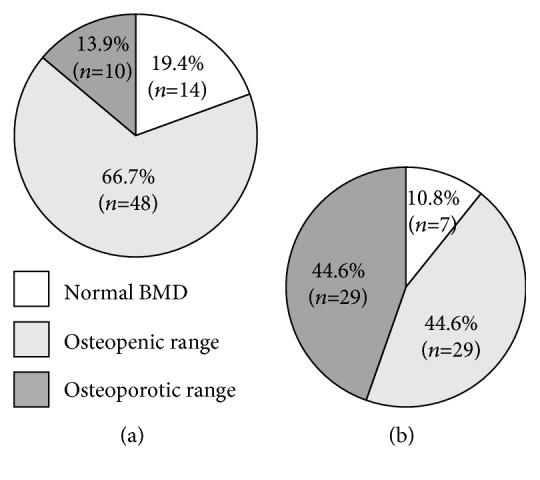
Prevalence of normal BMD and osteopenic/osteoporotic range for all major sites in the CKD G4 and CKD G5D subgroups. (a) CKD G4, *n* = 72 (54 men, 18 women). (b) CKD G5D, *n* = 65 (48 men, 17 women).

To address the question how the degree of renal impairment affects bone metabolism, the comparison of CKD G4 and CKD G5D subgroups was performed and anthropometric, laboratory, and densitometric characteristics of both groups are listed in Tables 3 and 4. BMD categories in CKD G4 and CKD G5D stages in comparison are listed in Figure 3 with significantly higher prevalence of osteoporosis in dialysed subjects (29/65 vs. 10/72; 44.6 vs. 13.9%; *p* < 0.001).

**Figure 4:**
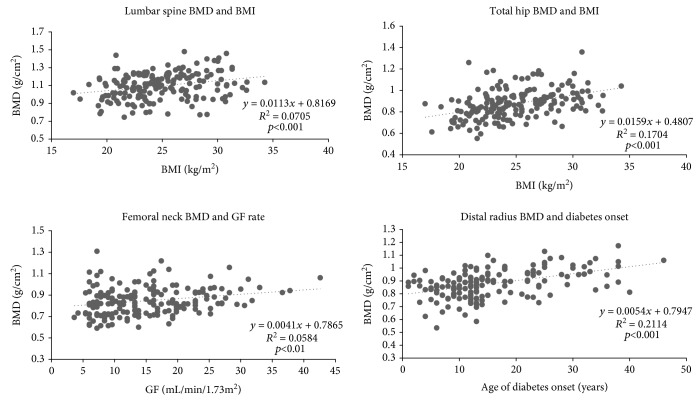
Selected correlations between BMD and anthropometric and laboratory parameters for the whole group.

Correlations between BMD in g/cm^2^ and selected anthropometric and laboratory parameters for the whole group and gender subgroups are listed in Table 5 and Figure 4. BMD correlated positively with age, BMI, daily insulin doses, and GF, but negatively with PTH and ALP. Correlations between age and DR BMD in all subjects (*r* = 0.27) and in men (*r* = 0.31), and between age and LS BMD (*r* = 0.32) in women, were significant even after adjustment for BMI (all with *p* < 0.05). The correlation between BMD and PTH was not significant after adjustment for GF.

Based on stepwise multiple regression analysis, independent predictors for BMD at all sites were age and BMI; for LS, it was PTH and for TH was discriminative GF. For FN, it was PTH in all cases and GF for men and, finally, for DR age of diagnosis across all subjects and ALP in men.

### 3.3. Treatment Analysis

With regard to bone-modifying therapy, some form of vitamin D was administered in 95 (53.7%) subjects: cholecalciferol substitution in 16 (9%), calcitriol in 75 (42.4%), and paricalcitol in 10 (5.6%) patients. Fifty-four (30.5%) patients were treated with a phosphate binder (calcium carbonate, sevelamer carbonate, or lanthanum carbonate), 28 (15.8%) with calcium medication including binders, and 4 (2.3%) subjects with cinacalcet. One-third of patients (58; 32.8%) were treatment-naive, a proportion comparable between men and women.

## 4. Discussion

We evaluated data concerning bone metabolism in patients with type 1 diabetes in advanced stages of diabetic nephropathy (CKD G4-G5) awaiting SPK. Overall, these subjects were considered at very high risk of bone metabolism impairment. However, the epidemiological data on osteoporosis and osteopenia prevalence were insufficiently precise.

We demonstrated an alarming prevalence of bone pathology, with 27.7% of subjects within the osteoporotic range and 56.5% within the osteopenic range, according to BMD measurement with DXA. Only 15.8% of subjects displayed normal BMD at all major sites, despite their relatively young average age. TH and FN were the most affected areas. We noted a gender difference in LS BMD, with significantly worse results in men. Age, BMI, and renal function level were the most important determinants of skeletal status.

The retrospective design of our study did not allow performing a direct comparison with a control group; therefore, we focused on available literature data about BMD values in type 1 diabetes and CKD subjects. The presence of type 1 diabetes is considered a strong risk factor for bone metabolism deterioration. Most studies describe at least a modest lowering of BMD in comparison to matched controls [1, 33]. Prospective studies evaluating changes in BMD in type 1 diabetes over time have not recorded any profound bone loss during the respective study periods [34–36]. However, all of the above studies concern patients with normal renal function. Bone impairment in terms of CKD-MBD starts to develop from the early stages of diabetic nephropathy onwards. The natural course of the disease in type 1 diabetes has yet to be described in detail. Clausen et al. [37] report a BMD decline in FN in men with type 1 diabetes, positive microalbuminuria, and normal kidney function. Even a GF value lower than 88.8 mL/min represents a risk factor for poor bone mineralisation in type 1 diabetes [3]. To our knowledge, studies of bone loss occurrence in CKD G3-G4 specific to diabetes have only evaluated older subjects (average age: 65 yr), mostly those suffering with type 2 diabetes. During one 2-year follow-up, not only did the average FN *T*-score decline from -1.88 to -2.07 but the prevalence of osteopenia doubled (from 25 to 52.5%) [38].

According to the NHANES study, the prevalence of osteoporosis in the general population with GF < 35 mL/min is 24% in women and 11% in men [39]. Recently, two Czech cross-sectional studies of similar Caucasian populations comprising subjects on haemodialysis or in the very early stages of hemodialysis treatment were published [40, 41]. The average TH *T*-score was -1.4 in both studies, while the average LS *T*-score was -0.5 and -0.3, respectively. We report an identical *T*-score for TH but a considerably lower *T*-score for LS, even though our study cohort was much younger (40 vs. 67 and 65 yr, respectively) and 41% of our subjects were already at stage G4 CKD. Subjects in the CKD G5D subgroup in our study displayed LS *T*-score -1.2 and TH *T*-score -1.7. Although both studies comprised subjects with diabetes (37% and 43%, respectively), they were not selected according to diabetes type, with type 2 diabetes patients presumably prevailing. An Australian group focusing on a similar cohort of mostly type 1 diabetes subjects awaiting SPK or kidney transplantation reported the following *Z*-scores for LS, FN, and DR: -0.15, -1.07, and -0.4, respectively [42]. In comparison with other solid organ recipients, the prevalence of osteoporosis among SPK candidates observed in our group was higher than that of heart failure patients (23%) and kidney failure subjects (24%) and lower than that of liver and lung transplantation candidates (31% and 67%, respectively) [43].

As stated above, the combination of long-lasting type 1 diabetes and progressive CKD has a significant negative impact on bone deterioration. The consequences of other diabetic complications, such as impaired vision, peripheral neuropathy, and diabetic foot, further reduce the physical capacities of patients, with increased inactivity leading to bone loss. Dietetic restrictions in CKD and diabetes (often complicated by diabetic gastroparesis) present added difficulties, including the likelihood of malnutrition, while the propensity for infections deepens the catabolic state. An association between diabetic retinopathy and lower BMD in FN was confirmed in a study by Campos Pastor et al. [44].

We observed that median *T*- and *Z*-scores were lower in TH and FN than in LS and DR. According to our review of the literature, cortical bone in the hips and radii of subjects with CKD G5 is more affected due to secondary hyperparathyroidism, while lumbar BMD tends to be closer to expected average values [45]. The worse results for lower limbs in our study might be explained by reduced physical activity. Moreover, the hip is the bone site most associated with reduced BMD in type 1 diabetes [2].

Contrary to expectations, men had significantly lower *T*- and *Z*-scores in LS than women, even after adjustments for age, BMI, and GF. The prevalence of osteoporosis at LS was almost two times higher in men. The median *T*- and *Z*-scores in women were close to normal. A study comparing gender differences based on densitometric results in a dialysed population confirmed worse results in women [46]. Although the pathophysiological mechanism behind our finding is unknown, some previously published studies regard male sex as an important risk factor for osteoporosis in type 1 diabetes. Hamilton et al. [47] report higher prevalence of osteoporosis in LS, and lower *T*- and *Z*-scores in LS, TH, and FN only in men in comparison with healthy controls. Hadjidakis et al. [48] report lower lumbar BMD only in men in comparison with healthy adults and lower FN BMD for both genders. A significantly higher percentage of osteoporosis in both LS and TH in men in comparison with women was reported in another study by Kemink et al. [49]. The above results apply to populations with normal renal function, which suggests that type 1 diabetes has a crucial effect on the bone.

Gonadal status was not systematically evaluated in our study. According to the literature, however, men with type 1 diabetes frequently display a certain level of hypogonadism [50], a condition that becomes even more pronounced upon development of CKD [51]. Premenopausal women with CKD and type 1 diabetes also face disturbances in the gonadal axis [52], but widespread use of hormonal contraception (HC) may mask these abnormalities. While 8 premenopausal women in our study (13%) reported recent or current use of HC, all 8 postmenopausal women (13%) reported no hormonal replacement therapy. Although the administration of HC is probably not the only explanatory factor, it may contribute to better results in LS bone density in women.

Surprisingly, patient age correlated positively with LS BMD in all subgroups and with TH and DR BMD in both the whole group and in men. Although this association can be partially explained by an increase in BMI in line with age, LS BMD in women and DR BMD in both the whole group and in men correlated with age even after adjustment for BMI. After reaching a plateau between the ages of 20 and 40, a gradual decrease in BMD follows. However, approximately half of the subjects in our study were younger than 40. Furthermore, a high proportion of our patients had most probably not reached their potential peak bone mass due to early diabetes onset. Subjects with progressive complications beginning in early childhood present for pretransplant investigation at a younger age and consequently have a higher bone mass deficit, a factor that might explain the correlation between age and BMD in our group. Again confirming our assumptions, LS, TH, and DR BMD in the men in our group correlated positively even with the age of diabetes onset.

BMD correlated strongly with BMI in all regions and across both genders, a finding in agreement with previously published data on HD subjects [53]. This correlation was clearly stronger in women, again indicating the importance of estrogen metabolism in adipose tissue for bone health. The association between TH, FN BMD, and GF confirms that good kidney function is crucial for cortical bone retention. PTH negatively correlated with LS, TH, and FN BMD, an observation that again tallies with previously published data [54, 55]. Total serum ALP correlated negatively with LS, FN, and DR BMD, again confirming previous findings [56, 57]. No association between glycosylated haemoglobin and BMD was detected. Daily insulin doses was associated with TH, FN, and DR BMD in the whole group and in men supporting its anabolic effect on the bone.

We confirm the general consensus that a high prevalence of vitamin D deficiency and insufficiency is typical in both T1DM [1] and CKD patients [58]. Only 6.1% of our subjects had normal levels, while calcitriol concentrations were adequate only in 31.3% of subjects. Deficits were diagnosed over the course of a whole year and even in subjects on substitution. Nevertheless, it should be noted that in some cases substitution was not indicated due to either hypercalcaemia, hyperphosphataemia, or suppressed levels of PTH. We did not demonstrate any significant relationship between current vitamin D levels and BMD.

A high percentage of subjects displayed hyperphosphataemia and PTH values outside the recommended range. The difficulty in reaching these target values, however, has been demonstrated by one large multinational observational study, with only 13.7% of its patients meeting the target KDIGO ranges for serum Ca, P, and PTH [59].

A clear limitation of our study is that our data were collected retrospectively. On the other hand, this reflects a common experience of clinical practice when processing unselected patients. We were restricted to analysing single laboratory values only. Likewise, we were unable to evaluate with sufficient complexity the overall standards for diabetes control, nutritional status, history, and burden of previous proteinuria or nephrotic syndrome or the progression of calcium-phosphate disorders. While there were differences in previous medical histories and treatments in our cohort, other clinically relevant causes of osteoporosis were rare. We encountered two cases of coeliac disease (albeit well-managed as part of a tailored diet), three cases of rheumatoid arthritis, one case of hepatitis granulomatosa, one case of liver cirrhosis, and four cases of subjects on antiepileptic drug therapy. Low-dose corticoid treatment applied to two women with systemic lupus erythematosus and ANCA vasculitis. Furosemide therapy was administered in 130 subjects and proton-pump inhibitor therapy in 39 subjects. Current smoking was documented in 42 patients, with 54 patients self-reported as ex-smokers.

Furthermore, we are aware that precisely diagnosing bone pathology in CKD based on discriminating “pure” osteoporosis from all other forms of CKD-MBD can only be established by biopsy analysis. In keeping with routine practice, however, we use the term osteoporosis for BMD within the osteoporotic range.

## 5. Conclusions

Bone metabolism impairment is frequently an overlooked complication of type 1 diabetes. In cases where combined with CKD-MBD serious bone loss follows as documented in our study with 27.7% of subjects categorised as having osteoporosis in any major site. All subjects who are intended to participate in transplantation programme in the future should be routinely screened since early stages of diabetic nephropathy and adequate preventive measures should be introduced.

Antithetical to the general consensus, we report that younger men with type 1 diabetes in advanced stages of CKD are at greater risk of having low bone density in lumbar spine than women. Further prospective studies to explain the etiology of gender difference in lumbar spine BMD decline are needed.

## Figures and Tables

**Table 1:** Anthropometric and laboratory characteristics for the whole group, men, and women, along with gender comparisons.

	Total (*n* = 177)	Men (*n* = 115)	Women (*n* = 62)	Significance
Age (yr)	40 [32.5 to 49]	44 [34 to 51]	36.5 [30 to 47]	*p* < 0.01
Diabetes duration (yr)	23 [18 to 30]	24 [18 to 31]	23 [18 to 29]	NS
Age of diabetes onset (yr)	13 [9.5 to 22]	14 [10 to 25]	12.5 [9 to 16.3]	*p* < 0.05
BMI (kg/m^2^)	24.2 [22.3 to 27.4]	25.0 [23 to 28.1]	23.0 [21.1 to 26.7]	*p* < 0.01
HbA1c (mmol/mol) (IFCC)	69 [63 to 82]	69 [62 to 82]	69.5 [63 to 81]	NS
(%) (DCCT)	8.46 [7.92 to 9.65]	8.46 [7.82 to 9.65]	8.51 [7.92 to 9.56]	
Daily insulin doses (IU)	39 [31 to 49]	41 [32 to 51]	34 [30 to 44]	*p* < 0.05
GF (mL/min/1.73 m^2^)	13.2 [8.4 to 19.8]	15.6 [9.6 to 20.4]	11.4 [7.8 to 18.0]	*p* < 0.05
CKD G4 (number)	72 (40.7%)	54 (47%)	18 (29%)	
GF (mL/min/1.73 m^2^)	21.0 [18.0 to 25.8]	21.6 [17.4 to 27.0]	21.0 [18.6 to 24.0]	NS
CKD G5 (number)	40 (22.6%)	13 (11.3%)	27 (43.5%)	
GF (mL/min/1.73 m^2^)	9.6 [7.8 to 13.2]	9.6 [8.4 to 13.2]	9.6 [7.8 to 12.6]	NS
CKD G5D (number)	65 (36.7%)	48 (41.7%)	17 (27.4%)	
GF (mL/min/1.73 m^2^)	8.4 [7.2 to 12.6]	10.2 [6.6 to 13.2]	7.8 [6.6 to 9.6]	NS
s-Ca (mmol/L) (*n* = 175)	2.39 [2.31 to 2.46]	2.4 [2.3 to 2.45]	2.38 [2.33 to 2.48]	NS
s-P (mmol/L) (*n* = 176)	1.58 [1.38 to 1.85]	1.54 [1.36 to 1.77]	1.62 [1.41 to 1.9]	NS
Alb (g/L)	32.9 [29.3 to 37.4]	34.4 [30.7 to 38.3]	30.6 [26.5 to 35.3]	*p* < 0.001
ALP (ukat/L)	1.48 [1.21 to 1.92]	1.58 [1.23 to 2.03]	1.37 [1.15 to 1.78]	NS
PTH (pmol/L) (*n* = 174)	15.3 [9.58 to 25.91]	15.1 [9.3 to 25.4]	15.9 [10.0 to 27.4]	NS
25OHD (ng/mL) (*n* = 148)	12.7 [10.4 to 19.5]	12.8 [10.4 to 20.1]	12.3 [10.3 to 18.0]	NS
1,25(OH)_2_D_3_ (ng/L) (*n* = 120)	14.9 [9.9 to 25.7] (+ 27 undetectable)	14.7 [10.1 to 25.8] (+ 15 undetectable)	15.2 [9.6 to 23.1] (+ 12 undetectable)	NS

Data are medians [interquartile ranges].

**Table 2:** Densitometric parameters for the whole group, men, and women, along with gender comparisons.

		Total (*n* = 177)	Men (*n* = 115)	Women (*n* = 62)	Significance
Lumbar spine	*T*-score	-0.9 [-1.85 to -0.05]	-1.1 [-2.0 to -0.2]	-0.4 [-1.6 to 0.33]	*p* < 0.01; *p*′ < 0.001
	*Z*-score	-0.8 [-1.75 to 0.1]	-1.1 [-2.0 to 0.0]	-0.3 [-1.33 to 0.33]	*p* < 0.001; *p*′ < 0.001
	BMD (g/cm^2^)	1.104 [0.982 to 1.211]	1.089 [0.976 to 1.196]	1.129 [0.987 to 1.222]	NS

Total hip	*T*-score	-1.4 [-2.0 to -0.9]	-1.5 [-1.9 to -1.0]	-1.4 [-2.23 to -0.7]	NS
	*Z*-score	-1.2 [-1.75 to -0.6]	-1.2 [-1.7 to -0.7]	-1.3 [-2.0 to -0.5]	NS
	BMD (g/cm^2^)	0.875 [0.789 to 0.946]	0.889 [0.823 to 0.963]	0.826 [0.742 to 0.917]	*p* < 0.01

Femoral neck	*T*-score	-1.6 [-2.3 to -1.0]	-1.7 [-2.3 to -1.0]	-1.5 [-2.3 to -1.0]	NS
	*Z*-score	-1.2 [-1.9 to -0.7]	-1.3 [-2.0 to -0.7]	-1.2 [-1.9 to -0.56]	NS
	BMD (g/cm^2^)	0.827 [0.745 to 0.928]	0.833 [0.776 to 0.943]	0.821 [0.724 to 0.903]	NS

Distal radius (*n* = 161)	*T*-score	-0.8 [-1.4 to -0.15]	-0.8 [-1.4 to -0.05]	-0.85 [-1.55 to -0.23]	NS
	*Z*-score	-0.8 [-1.4 to -0.1]	-0.7 [-1.4 to -0.05]	-0.8 [-1.4 to -0.2]	NS
	BMD (g/cm^2^)	0.890 [0.800 to 0.954]	0.926 [0.862 to 0.993]	0.813 [0.753 to 0.865]	*p* < 0.001

Data are medians [interquartile ranges]. *p*′, adjusted *p* for age (*T*-score) and for BMI, GF, and albumin (both *T*- and *Z*-scores).

**Table 3:** Anthropometric and laboratory characteristics for the CKD G4 and CKD G5D subgroups, along with their comparison.

	CKD G4 (*n* = 72) (54 M, 18 W)	CKD G5D (*n* = 65) (48 M, 17 W)	Significance
Age (yr)	42 [30.8 to 50]	40 [34 to 48]	NS
Diabetes duration (yr)	24 [19 to 31]	22 [17 to 30]	NS
Age of diabetes onset (yr)	13 [9 to 19.8]	13 [11 to 22.5]	NS
BMI (kg/m^2^)	24.7 [22.8 to 27.2]	23.9 [21.7 to 27.4]	NS
HbA1c (mmol/mol) (IFCC)	73 [65 to 84]	67 [57 to 82]	*p* < 0.05
(%) (DCCT)	8.83 [8.12 to 98.4]	8.28 [7.32 to 9.65]	
Daily insulin doses (IU)	41.0 [31.1 to 52.2]	38.0 [32.0 to 48.0]	NS
GF (mL/min/1.73 m^2^)	21.0 [18.0 to 25.8]	8.4 [7.2 to 12.6]	*p* < 0.001
s-Ca (mmol/L) (*n* = 136)	2.4 [2.32 to 2.46]	2.39 [2.3 to 2.5]	NS
s-P (mmol/L) (*n* = 136)	1.43 [1.27 to 1.58]	1.71 [1.48 to 2.05]	*p* < 0.001
Alb (g/L)	33.2 [29.8 to 37.4]	33.3 [30 to 38.4]	NS
ALP (ukat/L)	1.41 [1.14 to 1.84]	1.61 [1.23 to 1.91]	NS
PTH (pmol/L) (*n* = 135)	11.5 [7.6 to 16.6]	19.6 [11.7 to 29.6]	*p* < 0.001
25OHD (ng/mL) (*n* = 113)	12.8 [10.4 to 18.6]	12.7 [10 to 20.1]	NS
1,25(OH)_2_D_3_ (ng/L) (*n* = 92)	20.7 [12.1 to 29.9] (+ 5 undetectable)	12.5 [9.3 to 24] (+ 16 undetectable)	*p* < 0.05

Data are medians [interquartile ranges]. M: men; W: women.

**Table 4:** Densitometric parameters for the CKD G4 and CKD G5D subgroups, along with their comparison.

		CKD 4 (*n* = 72) (54 M, 18 W)	CKD 5D (*n* = 65) (48 M, 17 W)	Significance
Lumbar spine	*T*-score	-0.6 [-1.5 to 0.2]	-1.2 [-2.5 to -0.4]	*p* < 0.01
	*Z*-score	-0.5 [-1.48 to 0.3]	-1.1 [-2.4 to -0.35]	*p* < 0.01
	BMD (g/cm^2^)	1.123 [1.044 to 1.232]	1.077 [0.925 to 1.159]	*p* < 0.01

Total hip	*T*-score	-1.3 [-1.8 to -0.7]	-1.7 [-2.3 to -1.2]	*p* < 0.01
	*Z*-score	-1.1 [-1.6 to -0.6]	-1.4 [-2.15 to -0.9]	*p* < 0.01
	BMD (g/cm^2^)	0.903 [0.826 to 0.971]	0.857 [0.738 to 0.917]	*p* < 0.01

Femoral neck	*T*-score	-1.4 [-2.0 to -0.8]	-1.9 [-2.6 to -1.4]	*p* < 0.001
	*Z*-score	-1.1 [-1.68 to -0.3]	-1.6 [-2.2 to -1.0]	*p* < 0.001
	BMD (g/cm^2^)	0.858 [0.802 to 0.96]	0.818 [0.724 to 0.887]	*p* < 0.001

Distal radius (*n* = 126)	*T*-score	-0.8 [-1.6 to -0.2]	-0.9 [-1.6 to 0.0]	NS
	*Z*-score	-0.8 [-1.6 to -0.15]	-0.9 [-1.55 to -0.1]	NS
	BMD (g/cm^2^)	0.896 [0.822 to 0.96]	0.894 [0.791 to 0.971]	NS

M: men; W: women.

**Table 5:** Correlations between selected anthropometric, laboratory, and densitometric parameters for the whole group, men, and women.

	Lumbar spine BMD (g/cm^2^)			Total hip BMD (g/cm^2^)			Femoral neck BMD (g/cm^2^)			Distal radius BMD (g/cm^2^)		
	ALL	M	W	ALL	M	W	ALL	M	W	ALL	M	W
Age (yr)	0.2^b^	0.19^a^	0.33^c^	0.21^b^	0.19^a^	0.14	0.09	0.05	0.09	0.34^c^	0.37^c^	-0.04
DM onset (yr)	0.11	0.22^a^	-0.09	0.14	0.2^a^	-0.14	0.12	0.18	-0.08	0.45^c^	0.58^c^	0.03
DM duration (yr)	0.14	-0.01	0.41^c^	0.07	-0.05	0.29^a^	-0.04	-0.17	0.19	-0.06	-0.16	-0.08
BMI (kg/m^2^)	0.27^c^	0.17	0.49^c^	0.45^c^	0.29^b^	0.66^c^	0.39^c^	0.21^a^	0.64^c^	0.37^c^	0.26^b^	0.38^b^
Insulin doses (IU)	0.09	0.12	0.05	0.30^c^	0.31^c^	0.16	0.31^c^	0.29^b^	0.27^a^	0.33^c^	0.32^b^	0.22
s-Ca (mmol/L)	0	0	0	-0.02	-0.05	0.01	0	-0.05	0.08	-0.16^a^	-0.18	-0.08
s-P (mmol/L)	0.11	-0.03	-0.29^a^	-0.15^a^	-0.17	-0.1	-0.13	-0.13	-0.1	-0.1	-0.17	0.13
PTH (pmol/L)	-0.2^b^	-0.15	-0.3^a^	-0.18^a^	-0.11	-0.24	-0.19^a^	-0.18	-0.2	-0.1	-0.14	-0.09
GF (mL/min/73 m^2^)	0.12	0.04	0.37^b^	0.23^b^	0.17	0.29^a^	0.24^b^	0.19^a^	0.29^a^	0.15	0.07	0.1
ALP (ukat/L)	-0.26^c^	-0.27^b^	-0.22	-0.13	-0.17	-0.23	-0.18^a^	-0.16	-0.28^a^	-0.13	-0.25^b^	-0.11
25OHD (ng/mL)	0.04	0.03	0.1	0.15 (*p* = 0.07)	0.17	0.13	0.15 (*p* = 0.07)	0.12	0.24	0.14	0.13	0.29

ALL: all subjects; M: men; W: women; ^a^*p* < 0.05; ^b^*p* < 0.01; ^c^*p* < 0.001.

## Data Availability

The data used to support the findings of this study have not been made available because of medical secrecy.
